# Implementation Intentions as a Strategy to Increase the Notification Rate of Potential Ocular Tissue Donors by Nurses: A Clustered Randomized Trial in Hospital Settings

**DOI:** 10.1155/2014/921263

**Published:** 2014-07-15

**Authors:** Frédéric Douville, Gaston Godin, France Légaré, Marc Germain

**Affiliations:** ^1^Institut Universitaire de Cardiologie et de Pneumologie de Québec, Québec, QC, Canada G1V 4G5; ^2^Faculty of Nursing, Laval University, Québec, QC, Canada G1K 7P4; ^3^Department of Family Medicine and Emergency Medicine, Laval University, Québec, QC, Canada G1K 7P4; ^4^Héma-Québec, Québec, QC, Canada G1V 5C3

## Abstract

*Aim.* The purpose of this study is to evaluate the impact, among nurses in hospital settings, of a questionnaire-based implementation intentions intervention on notification of potential ocular tissue donors to donation stakeholders. *Methods.* This randomized intervention was clustered at the level of hospital departments with two study arms: questionnaire-based implementation intentions intervention and control. In the intervention group, nurses were asked to plan specific actions if faced with a number of barriers when reporting potential ocular donors. The primary outcome was the potential ocular tissue donors' notification rate before and after the intervention. Analysis was based on a generalized linear model with an identity link and a binomial distribution. *Results.* We compared outcomes in 26 departments from 5 hospitals, 13 departments per condition. The implementation intentions intervention did not significantly increase the notification rate of ocular tissue donors (intervention: 23.1% versus control: 21.1%; *χ* ^2^ = 1.14, 2; *P* = 0.56). *Conclusion.* A single and brief implementation intentions intervention among nurses did not modify the notification rate of potential ocular tissue donors to donation stakeholders. Low exposure to the intervention was a major challenge in this study. Further studies should carefully consider a multicomponent intervention to increase exposure to this type of intervention.

## 1. Introduction

In many countries, the demand for ocular tissue donation exceeds the supply, leaving a shortage [1–4]. Despite donation promotion campaigns, the number of donated ocular tissues is still lower than the demand. For instance, in the United Kingdom, 2000 to 3000 cornea transplantations are performed per year, but additional 500 corneas would have been needed [1, 5]. Similarly, in France 4500 corneas are grafted per year, but approximately 7500 persons are still awaiting transplantation [4]. The situation is similar in Canada where approximately 20 000 ocular tissues are transplanted per year among 50 000 persons on the waiting list [6]. It is nonetheless important to note that some of these countries import foreign corneas to supply the demand [3] which make it difficult to truly estimate the real ocular tissue shortage.

In an opt-in donation regulation system such as that prevailing in Canada [7], organ and tissue donation is based on family acceptance and the detection by health professionals who then approach families to obtain donation consent. Thus, part of the tissue shortage might be attributable to a lack of notification of potential donors by health professionals (nurses and physicians) [8–10], despite their favourable attitudes and intentions towards organ and tissue donation [5, 11–18]. The role of health professionals regarding organ and tissue donation process is to identify potential donors, to seek consent for organ and tissue donation, to approach patient's next-of-kin, and to notify donation stakeholders [19] such as organ procurement organizations (OPO), OPO specialists, or tissue banks. OPO specialists have been introduced in many clinical settings in order to increase the number of organ donors [20]. However, this strategy has yet to improve the situation, and the number of tissue donations remains low or insufficient to meet Canadian needs. One reason might be that OPO and procurement organizations must be notified by health professionals (nurses and physicians) of potential tissue donors. This suggests that in an opt-in regulation system, more effort is needed to help and support professionals, such as nurses, in the notification of potential donors.

Implementation intentions have been suggested by Gollwitzer as an effective strategy to increase the adoption of a given behavior when intention (person's degree of motivation to act) is favorable at baseline [21, 22]. This process implies to consciously act by associating a mental representation of a given situation and the means to achieve these goals or perform targeted behaviors [23]. The implementation intentions give a voluntary control to an individual to plan to perform a certain behavior when a specified condition is met [21]. However, implementation intentions rest on the premise that an individual original intention is positive towards the targeted behavior. This theoretical approach cannot generate the adoption of a behavior from persons with negative intentions to perform this specific behavior. Since health professionals have favourable intentions towards organ and tissue donation [5, 11–18], an implementation intentions intervention should increase the notification of potential donors. The implementation intentions strategy [21] is a theory-based behavioral change approach involving the enactment of the individual's intention to adopt the action or behavior. This strategy encourages individuals to adopt the desired behavior when a specific situation is met [21], such as notifying donation stakeholders for every potential ocular tissue donor. Implementation intentions can take the form of an “if-then” plan linking critical situations: (“if”) with appropriate behavioral responses; (“then”) to achieve the desired goal [21, 23–26]. Thus, engaging in the formulation of an action plan allows individuals to make use of strategic environmental signals and act effectively [21, 27]. Therefore, the people who make action plans are more likely to act in the expected management [21] and adopt the targeted behavior more quickly [28] than those who do not form a plan [27]. Notwithstanding earlier observations on its effectiveness among health professionals [24, 29], most previous studies using the implementation intentions strategy were performed among small samples (*n* = 78 and 182 health professionals, resp.) using multicomponent interventions (continuing education class or workshop followed by booster sessions). It remains to be verified if this technique could be used effectively in larger groups of nurses working across multiple clinical sites, using a single and brief intervention rather than a multicomponent intervention.

The purpose of this study was to develop, implement, and evaluate the impact, among nurses in hospital settings, of a questionnaire-based implementation intentions intervention aimed at increasing the notification of potential ocular tissue donors to donation stakeholders.

## 2. Materials and Methods

### 2.1. Design

We performed an experimental study, clustered at the level of hospital departments with two study arms: questionnaire-based implementation intentions intervention and control. To avoid potential bias caused by the distribution of two different questionnaires (experimental and control) in a given department and because notification rates are available only by department rather than for individual health professionals [30, 31], cluster randomization of departments was performed.

### 2.2. Participants

Twenty-seven departments from five hospitals participated in this intervention study. These departments were chosen because they were likely to encounter ocular tissue donation, for example, emergency departments, intensive care units, or palliative care units. Thereby, outpatient clinics or administrative departments were excluded. Also, all the selected departments were operated in clinical settings where OPO representatives coordinate donation. This criterion ensured that nurses in these departments had some knowledge of organ and tissue donation at the onset of the study, since OPO representatives offer regular support to nurses, heighten their awareness of the donation process, and help them approach families.

### 2.3. Randomisation Procedure

Given that the notification rate varied between departments and was relatively low (mean rate around 15% two years before the intervention), departments were paired according to their baseline notification rate. The two departments with the highest rates were paired together, then the next two highest, and so on. The department' notification rate varied from approximately 25% for the highest department pairing to 0% for the lowest department pairing. Each pair was then divided and randomly assigned to the intervention or control group.

### 2.4. Intervention

The intervention consisted of a self-administered questionnaire querying nurses in the experimental departments about their intentions to notify potential ocular tissue donors (3 items) and inviting them to plan specific actions “if” faced with a number of barriers to notify potential ocular tissue donors. These barriers were based upon a literature review and OPO experience (examples: lack of time to notify, feeling uncomfortable to approach a family, never approached a family, fear of family reaction, lack of knowledge, etc.). In this intervention, the items corresponding to the action plan were developed based on Gollwitzer's implementation intentions strategy [21]. The use of a paper-based questionnaire corresponds to a single and quick implementation intentions intervention rather than multicomponent interventions already studied in the past. This questionnaire took a maximum of 5 minutes to complete, allowing nurses to finish it easily during a working day (lunch or day breaks).

The intervention instrument was tested and validated by three experts in measurement and behavioral sciences (evaluating the relevance and clarity of the questions, instructions, and answer options) before its use. Questionnaires were also adjusted based on the recommendations of these experts.

Participants in the control group received another questionnaire that did not include the implementation intentions strategy. The control group questionnaire only asked questions regarding nurses' intention to notify potential ocular tissue donors (3 items).

For administrative reasons, the distribution strategy of the intervention questionnaires for each department was planned by the head nurses of the five hospitals. They requested that boxes of questionnaires be left at each department where supervisors invited nurses to complete the questionnaire during the intervention period (November 2010). Nurse supervisors were blinded in their department's randomization group. After completing the questionnaire, the nurses were invited to return it anonymously by internal mail to the principal investigator's office.

### 2.5. Sample Size

We estimated the sample size based on the mean baseline notification rate of potential ocular tissue donors: 15% two years before the intervention in the five hospitals participating in the study. We assumed that the minimum clinical significance for an increase in notification rate of potential ocular tissue donors after the implementation intentions intervention would be 10%. Assuming a possible 5% participation-related increase in the control group notification rates to a “question-behavior” (mere-measurement) effect, we aimed to detect a 15% increase in the intervention group. This notification rate increase would be clinically significative and represent for many departments an increase ranging from 2 to 5 potential ocular tissue donor notifications. To detect a possible increase from baseline 15% to 30% with 80% power at a 5% significance level and a small correlation of 0.10 between the pre- and postintervention rates, we needed 398 potential donors per group for a total of 796 donors. To achieve this number of potential donors, data over a six-month period were required.

### 2.6. Primary Outcome

The primary outcome of this intervention study was the ocular tissue donors' notification rate for each department. The ocular tissue donors' notification rate is expressed as the ratio between the number of potential ocular tissue donor notifications and potential number of ocular tissue donors. Monthly data on the potential number of ocular tissue donors (all deceased patients that were 85 years old or less and not presenting systemic infection) during the observation period were first obtained for each department from the archives of each hospital. The achieved number of tissue donors' notifications was obtained from the database of the provincial tissue bank for each department during each month of the study period. It should be noted that all notifications were counted, not just those that resulted in an actual donation.

Intention to notify potential ocular tissue donor was evaluated with three items. The aim was to assess the basic assumption of implementation intentions; an individual original intention must be positive towards the given behavior.

### 2.7. Data Collection

Measurement of potential and achieved numbers of ocular tissue donors was planned to take place six months before and six months after the intervention between May 2010 and May 2011 to evaluate a time and group effect. However, the follow-up period had to be shortened because the Ministry of Health introduced a legislative change regarding organ and tissue donation that made mandatory the notification, by clinical settings, of all potential organ and tissue donors to donation stakeholders. This regulation was implemented halfway through the follow-up period. Therefore, the follow-up period was ended at three months instead of the planned six months.

### 2.8. Ethical Considerations

This study received approval from the research ethics committee of the two institutions regrouping the five hospitals.

### 2.9. Data Analysis

Statistical analysis was performed by a statistician who was blinded to the implementation intentions questionnaire intervention distribution. The primary outcome was assessed by comparing the difference between the experimental and the control groups in mean ocular tissue donors' notification rates before and after the intervention. Results are reported at the level of departments. Ocular tissue donors' notification rates were analyzed using a generalized linear model with an identity link and a binomial distribution. The model included a group effect (control versus intervention), a time effect (preintervention and postintervention), and a “time × group” interaction effect. The effect of the intervention was assessed using the interaction term. Generalized estimating equations were used to account for the correlation in time. Analyses were executed with SAS version 9.2 using a bilateral level of significance of 5%.

## 3. Results

**Figure 1:**
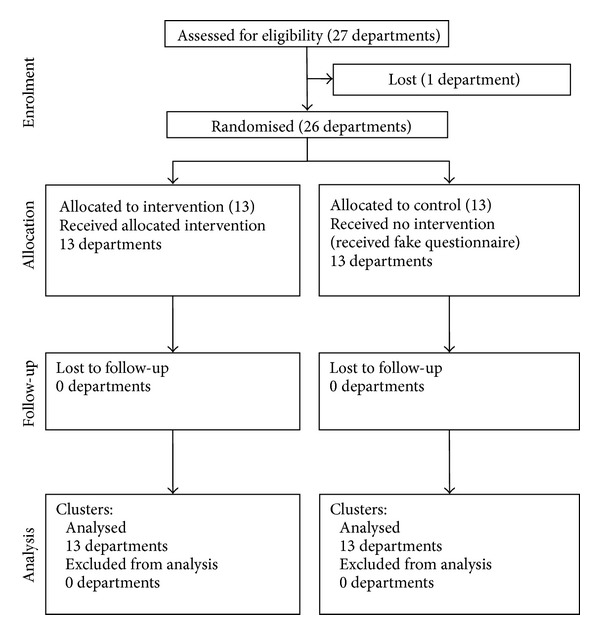
Diagram flow of clusters.

In total, 26 of the 27 departments participated in the study (Figure 1). One department in the experimental group withdrew before the intervention period due to a change in vocation; this department became an outpatient clinic and was excluded from statistical analyses, keeping intention to treat analysis only. The departments in the intervention and control groups had a mean of, respectively, 56 and 46 nurses, ranging from 11 nurses (palliative care unit) to 127 nurses (intensive care units). A total of 1341 nurses were invited to complete a questionnaire (intervention and control).

The intervention revealed a very low level of exposure: only 9% of the nurses returned the intervention questionnaire, compared to the 27% of nurses who returned the control group questionnaire. The intention to notify potential ocular tissue donor by nurses in the intervention and control group was similar and, respectively, 5.6 ± 1.4 and 5.7 ± 1.6 on a 7-point Likert scale (where 1 represented a poor intention and 7 a high intention). Ocular tissue donors' notification rates calculated for the six-month period before and the three-month period after the intervention are presented in Table 1.

Contrast results for generalized estimating equation analysis showed no statistical difference between the control and intervention groups before the intervention (*χ* ^2^ = 0.09, *P* = 0.76), for they both had similar notification for the six-month period before the study (resp., 21.1% and 23.1%). The notification rate remained similar for the control group before and after the intervention period (*χ* ^2^ = 0.00, *P* = 0.97). Also, the intervention group did not show a statistical difference in the notification rate compared with the control group during the study period (*χ* ^2^ = 0.08, *P* = 0.78).

## 4. Discussion

Our findings show that a questionnaire-based implementation intentions intervention among a group of nurses did not significantly increase the notification rate of ocular tissue donors in the experimental group.

In the present study, in the six-month period before the study, the notification rate was 22.1% overall. This means that donation consent would not be obtained for nearly 80% of potential donors, or that notification of potential donors would not be performed by healthcare professionals. This rate was consistent with eye donation rates in other studies in different clinical settings (between 23% and 40%) [1, 32]. Obviously, given the gap between supply and demand in cornea donation, major gains are yet to be recorded.

Although there are many interventions aimed at changing health professionals and nurses behavior towards organ and tissue notification in clinical settings, only a few have been carried out exclusively among healthcare professionals whose job position requires them to be in contact with patients and who are in a position to ask for donation consent [33, 34]. Indeed, most interventions target hospital administrators, clerical staff, and chaplains [34–37]. As such, it is difficult to isolate the impact of those interventions on nurses' behavior.

The lack of studies assessing the behavior changes or health outcomes in the literature is consistent with a recent publication that reviewed the evaluation of interprofessional education programs. According to Kirkpatrick's levels [38], only 9.7% of program evaluations assessed changes in behavior, 0.004% looked at organizational practice changes, and no items addressed benefits to patients [39]. Similar results were obtained in continuing nursing education programs [40].

We chose to develop this theory-based intervention in order to fulfill the absence of theoretical basis in previous interventions in this domain [41, 42]. Since nurses are known to have favourable intentions regarding donation [11, 12], it was expected that this type of intervention would have improved ocular tissue donors' notification. Indeed, Gollwitzer's implementation intentions strategy [21] is known to be efficient and has already been proven effective in studies among health professionals [24, 29]. However, such was not the case in the present study. A number of reasons might explain this situation: low exposure to the intervention, incorrect task performance in the intervention group, mere-measurement effect, and an unexpected change in legislation.

First, the single and brief intervention adopted in this study led to a very low level of exposure to the intervention. Less than 10% of nurses in the intervention group completed and returned the questionnaire. Given the nature of the anticipated effect size, this level of exposure to the intervention did not achieve the required standard. This observation suggests that it might be questionable to use this survey technique in studies carried out in clinical settings. Nurses are confronted with an increased workload and are unlikely to be available to complete questionnaires during working hours, even though the intervention questionnaire could be completed in less than five minutes. However, if this survey technique is the only means of contact with health professionals in clinical settings, it should be explored to adopt a multicomponent intervention using additional survey methods such as prenotification letters, reminders, and incentives [43].

Secondly, because the study adopted a self-administered questionnaire, it was impossible to ascertain the degree to which the nurses in the experimental group performed their implementation intentions task correctly (planned specific actions “if” faced with a number of barriers to notify potential ocular tissue donors). For instance, some nurses might be embarrassed about death, so they might not have been comfortable answering a questionnaire about their notification behavior when confronted with a dying patient, or they might have been less likely to implement their notification behavior because of this discomfort [5].

Thirdly, the control group was also exposed to an intervention, since they were also asked to complete a questionnaire (excluding implementation intentions questions) and since a “question-behavior” (mere-measurement) effect for this type of questionnaire has been reported in the past [44, 45]. However, since both the control group and the experimental group did not show an increase in the notification rate, it is unlikely that this effect occurred in the present study. Nonetheless, blinding participants in the allocation group using a “placebo” questionnaire remains essential to distinguish between potential questionnaire effect and implementation intentions effect.

Finally, the observation period was halved due to a provincial legislative change. The modification of Quebec Bill 125 now requires mandatory notification in clinical settings of all potential organ and tissue donors to donation stakeholders. Consequently, our intervention may have become useless, assuming that all clinical settings would have complied with this mandatory notification. Thus, the postintervention period was shortened from six to three months. The reduction of the follow-up period negatively affected the power of our study, since only the situation of 396 of the 796 potential donors was documented. Consequently, the power of the study was inadequate to detect a change in notification rate. Curtailing the study was, nevertheless, the optimal strategy in the present case, to ensure methodological rigour.

### 4.1. Limitations

The major limitations in this study are low exposure to the intervention and insufficient power. These limitations were addressed earlier in Section 4, since they explain why Gollwitzer's implementation intentions strategy was not efficient in this study.

## 5. Conclusion 

In conclusion, a questionnaire-based implementation intentions intervention among nurses did not change the notification rate of potential ocular tissue donors to donation stakeholders. However, it is not possible at this point to state that the implementation intentions approach is inappropriate, since the study had to be shortened due to an unexpected legislative change leading to mandatory notification of potential donors. Likewise, an insufficient proportion of nurses in the experimental group were exposed to the intervention. Low exposure due to low questionnaire response rate is a major challenge when using a single and brief implementation intentions intervention among nurses in hospital settings. Further studies should carefully consider adding specific strategies such as a multicomponent intervention to increase exposure to this type of intervention.

## Figures and Tables

**Table 1:** The number of donors and notifications per group and period.

Periods^1^	Control (*n* = 13 departments)				Intervention (*n* = 13 departments)			
	Referred donors (*n*)	Potential donors (*n*)	Notification proportion (%)	CI	Referred donors (*n*)	Potential donors (*n*)	Notification proportion (%)	CI
Pre	83	390	21.3	11.1–31.4	73	316	23.1	12.1–34.1
Post	52	246	21.1	7.5–34.8	37	150	24.7	14.2–35.1
Difference^2^	−31	−144	−0.2	−7.0–6.7	−36	−166	1.6	−4.7–7.8

^1^Pre- and postintervention periods spanned over 6 and 3 months, respectively.

^2^Post- and predifference. *P* value of the group-by-time interaction testing if the post- and predifference are equal in both groups = 0.78.
